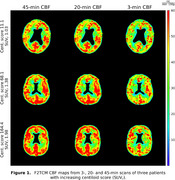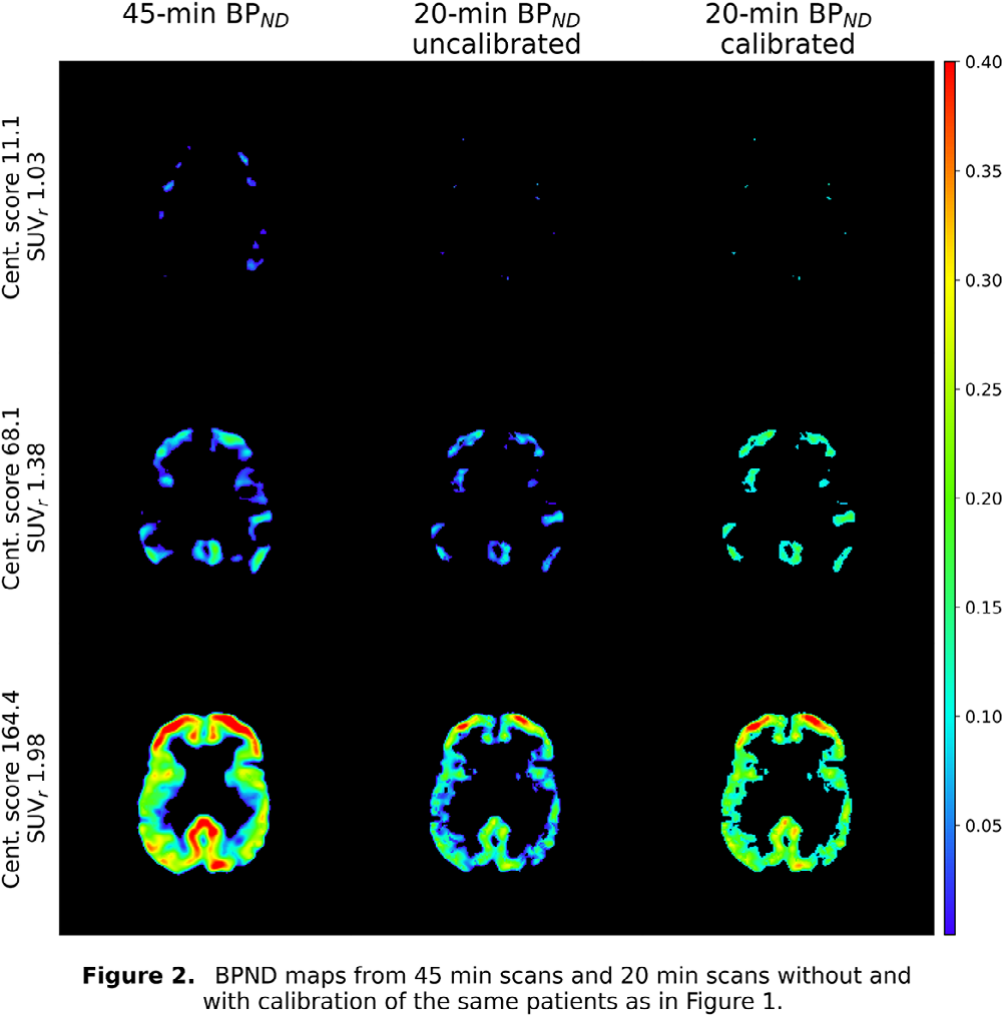# Simplified Workflow using a 20 min Dynamic 18F‐Florbetaben PET Scan to Measure Cerebral Blood Flow and Binding Potential

**DOI:** 10.1002/alz70856_097880

**Published:** 2025-12-24

**Authors:** Danny De Sarno, Jaspreet Bhangu, Michael J Borrie, Ting‐Yim Lee

**Affiliations:** ^1^ Western University, London, ON, Canada; ^2^ St. Joseph's Health Care London, London, ON, Canada

## Abstract

**Background:**

PET imaging plays a critical role in the diagnosis and follow‐up of Alzheimer's disease (AD). However, current methods face significant limitations for two key measures: (1) Non‐displaceable binding potential (BP_ND_) of amyloid‐beta (Aβ), which measures Aβ plaque accumulation but requires prolonged >100 min study times; (2) Cerebral blood flow (CBF), an indicator of neurodegeneration which typically requires an additional, separate study. To address these challenges, we developed **a novel processing workflow which calculates absolute CBF from standard dynamic Aβ PET scans using our lab's flow‐modified 2‐tissue compartment model (F2TCM,** EJNMMI Res.11:2**), and estimates BP_ND_ from a 20‐min dynamic scan post‐injection through multiple‐fold cross‐linear calibration**.

**Method:**

Data from 10 patients enrolled in the ongoing BioMind clinical trial data at our institution were used. Each dataset included a 45 min dynamic scan after injection of 300 MBq of 18F‐florbetaban and a 10 min scan at 110 min post‐injection, acquired with a GE Healthcare OMNI Legend PET/CT scanner. Images were reconstructed using the Q. Clear protocol for higher resolution arterial input function measurements, and the smoother VPHD protocol to improve signal‐to‐noise for CBF estimation. CBF was calculated using the F2TCM, BP_ND_ using Logan graphical analysis relative to the cerebellum, and centiloid scores and SUVr were obtained with MIMneuro (MIM Software Inc.).

**Result:**

Figure 1 shows the similarities/differences of CBF maps derived from 3‐, 20‐ and 45‐min dynamic scans for three patients with 18F‐florbetaben centiloids (SUVr) of 11.1 (1.03), 68.1 (1.38), and 164.4 (1.98), respectively. Voxel‐wise comparisons of CBF of all 10 patients showed mean MSE of 18±11 and 156±110 mL/min/100g for 20 and 3 min relative to 45 min, respectively. Figure 2 shows BP_ND_ maps for a 45 min scan, followed by uncalibrated and calibrated 20 min scans. Calibration slopes, intercepts, uncalibrated and calibrated MSEs were 0.95±0.03, 0.08±0.01, 0.012±0.0044 and 0.0070±0.0013, respectively, based on five‐fold cross calibration.

**Conclusion:**

This study highlights the potential of a streamlined 20‐minute Aβ PET imaging protocol to measure BP_ND_ as a surrogate for the centiloid score, while also providing complementary CBF measurements to enhance diagnostic and prognostic utility.